# Health care utilisation of asylum seekers and refugees in the South-West of Germany

**DOI:** 10.1371/journal.pone.0299886

**Published:** 2024-04-18

**Authors:** Annabelle J. Bockey, Cornelia Braun, Johannes Camp, Aleš Janda, Winfried V. Kern, Anne-Maria Müller, Katarina Stete, Siegbert R. Rieg, Berit Lange

**Affiliations:** 1 Department of Medicine II, Division of Infectious Diseases, Medical Centre—University Hospital Freiburg, Faculty of Medicine, Freiburg, Germany; 2 PhD Programme “Epidemiology” Braunschweig-Hannover, Helmholtz Centre for Infection Research, Braunschweig, Germany; 3 Clinic for Refugee Medicine, Medical Centre–University of Freiburg, Faculty of Medicine, Freiburg, Germany; 4 Centre for Paediatrics and Adolescent Medicine, Medical Centre–University of Freiburg, Faculty of Medicine, Freiburg, Germany; 5 Department of Paediatrics and Adolescent Medicine, University Medical Centre Ulm, Germany; 6 Centre for Mental Health, Department of Psychosomatic Medicine and Psychotherapy, Medical Centre–University of Freiburg, Faculty of Medicine, Freiburg, Germany; 7 Helmholtz Centre for Infection Research, Braunschweig, Germany; National Institute of Public Health: Instituto Nacional de Salud Publica, MEXICO

## Abstract

**Background:**

Limited evidence on utilisation of health care by recently arrived asylum seekers and refugees in high-income countries is available. This study aims to describe the implementation of an integrated care facility (ICF) in an initial reception centre and measure the utilisation of care and the influence of operational parameters.

**Methods:**

In a retrospective cohort study design, using medical records, we followed inhabitants of a reception centre in Germany between 11.10.2015 and 30.05.2018. We assessed frequency of visits and revisits to a newly established integrated care facility (ICF), and the effects of the ICF on visits to the local emergency department (LED) in the regional tertiary hospital using survival analysis and time series regression. We also explore the influence of operational parameters on the different implementation phases; phase 1: provisional clinic with 1–2 hours of physician presence daily, phase 2: implementation of ICF with 2–4 hours of care by a team of doctors and nurses daily, phase 3: routine running of ICF with daily operational hours of 10am–2pm with care provided by an interdisciplinary team of doctors and nurses.

**Results:**

14,419 total medical visits were recorded from 1,883 persons seeking health care in the ICF. The absolute number of visits per day remained similar over the study period (19·9/day), yet the relative number of visits changed from 2·2 to 15 per 100 inhabitants from phase 2 to 3, respectively. Most visits were due to respiratory infections (612/3080, 20%), and trauma and musculoskeletal conditions (441/3080, 14%). The rate of revisits to ICF was 2·9 per person per month (95%CI 2·9–3), more for those older, female, from North Africa and those with a translator present. The ratio of visits to the LED changed from 0·3/100 inhabitants per day to 0·14/100 inhabitants after implementation of the ICF and back to 0·3/100 inhabitants during the routine running.

**Conclusions:**

Though seasonal variation and referral practices must be considered, a high rate of revisits to the ICF were recorded. While visits to the LED decreased after the implementation of the ICF, visits returned to the pre-ICF levels during the routine running of the ICF. The results show that AS&R require reliable access to health care, yet the needs of specific groups of migrants may be different, especially those with language barriers, minority groups or those from certain regions. As such, care should be migrant sensitive and adapt to the changing needs of the population. Though more research is required to better understand the differing needs of migrants, this study may help to inform guidelines surrounding migrant sensitive standards of care in Germany.

## 1. Introduction

Since 2012 a now well described population movement to central and western Europe took place, with particularly high numbers of people arriving to Germany and Sweden between 2014 and 2016. While there is a long standing discussion on whether centralised centres should be built to accommodate newly arriving asylum seekers [1], and if so for what duration [2], most regions have adopted this approach in the past with rapidly changing numbers of incoming asylum seekers [3]. Despite this, recent studies have shown that accommodation characteristics have a considerable influence on the health of those staying within these settings, and worse outcomes are notable for high occupant settings and more remote accommodation[4].

In Germany, newly arriving asylum seekers spend several weeks to several months in temporary shelters, often known as reception centres. By law, asylum seekers are housed in reception centres from arrival until a decision is reached on their application for asylum. However, sometimes transfers to other housing may be delayed regardless of whether they have been officially acknowledged as a refugee. For this reason, in this paper, we will refer to the population staying in reception centres as asylum seekers and refugees (AS&R). While housed in reception centres, the movement of resident AS&R are significantly limited, with imposed curfews, only residents and restricted personnel permitted in the grounds, and strict incoming and outgoing checks. Food is usually provided on site, while some centres additionally offer social services, childcare and medical support.

Screening for active tuberculosis is normally performed in reception centres, yet delivery of further health care varies greatly between different cities and regions. Officially, under the Asylum Seekers’ Benefits Act, the German law restricts free health care provision to asylum seekers within their first 18 months in Germany to: necessary medical or dental treatment for acute health issues, necessary preventative medical check-ups, and additional special provisions for pre- and post-natal care to expectant or new mothers [5]. Often when seeking care, asylum seekers must first obtain a health insurance voucher provided by medical personnel in the reception centres, a practice that is intrinsically flawed and leads to disadvantages for AS&R accessing health care [6, 7]. Consequently, some Federal States and municipalities have issued asylum seekers ‘normal’ health insurance cards, which has been shown to reduce such disadvantages and increase use of specialist services [6].

During 2015, this required ubiquitous adaptive measures of health care systems. Modifications in Germany took place on a health system level in response to the publication and implementation of screening guidelines [8–10]. However, the creation of these guidelines lacked tangible evidence on disease epidemiology in this population and anticipated a rapid rise in less familiar infections, which we now know is more similar to usual disease spectrum recorded locally, apart from increased incidences of tuberculosis, some infections more common in tropical regions [11], and mental health concerns [12]. Subsequently, local health care systems and institutions had to adapt to a changing epidemiology and a “new” population group. A significant proportion of this adaptation effort was initially provided by civil society, non-government organisations and volunteers [13]. Institutional adaptations to the first arrivals were common, and some of these have now been described [14, 15].

Adaptability of health care systems and local health care protects health and society and has been identified as a key issue in the building of resilient health care systems [16]. This has been well described for health care systems in low- and middle-income countries for climate change and population movement [17, 18]. However, quantified evaluation of adaptive measures in high-income countries are rare and description of the necessary resources and quantitative effects of adaptation efforts in local health care systems are scarce [19, 20]. This makes it difficult to implement adaptive measures, which is why more evidence on such efforts is needed, particularly for the first arrival situation of AS&R in high-income countries [19].

In this study, we describe here the organisation, required resources and effects in terms of health care usage at the facility, and at nearby medical facilities, of an integrated care facility (ICF) that was implemented in the main initial reception centre (FIRC) during 2015 and 2016 in Freiburg, Germany [21]. During the initial planning of the integrated health model in 2015 for incoming AS&R in the South-Western German city, evidence of usage and effect of different health care models was not available, and the provision of resources were either approximations by implementing institutions or random due to the availability of volunteer personnel. We believe that the publication of these parameters can help to build evidence on establishing models for the health care of incoming AS&R, often under rapidly changing circumstances, in high-income countries when centralised first arrival options are implemented.

## 2. Material and methods

### 2.1 Reporting guidelines

We followed STROBE reporting guidelines for observational studies [22].

#### 2.2.1 Setting

This study took place in the Freiburg initial reception centre (FIRC) with a primary capacity of 1,000 persons located in the city of Freiburg: a city of 230,000 inhabitants in the state of Baden-Wurttemberg in Germany. The FIRC was one of firstly three initial reception centres in the state that were rapidly built up to provide accommodation for incoming AS&R during 2015, which were added to the already existing centres in Karlsruhe and Heidelberg. Due to logistical and infrastructure challenges the centre was set up in September 2015 with two tents and was only moved into existing buildings in 2018. Administration of these centres is the responsibility not of the city but of the regional administration (Regierungspräsidium).

The centre is placed within Freiburg city and is well connected by tram and bus with the city centre and local businesses, institutions and health care providers. At the time of the study, it contained two large tent structures with compartments within for individual beds. The centre accommodated AS&R families and individuals, yet unaccompanied children were housed elsewhere. Within the initial reception centre there is an “Infopoint” that directs inhabitants to needed resources, social workers, a cafeteria, playground and day-care for young children and bathroom facilities.

Although the state of Baden-Wurttemberg was not included in the group of Federal States that issued resident asylum seekers with ‘normal’ health insurance cards, medical care was planned for the FIRC. Originally only one to two hours of doctor presence was planned for this centre with additional referral to local health facilities. After it became clear that this was insufficient, an interdisciplinary group from the university hospital offered to build a model that would provide health care to inhabitants of the reception centre.

#### 2.2.2 Integrated care model

**Fig 1:**
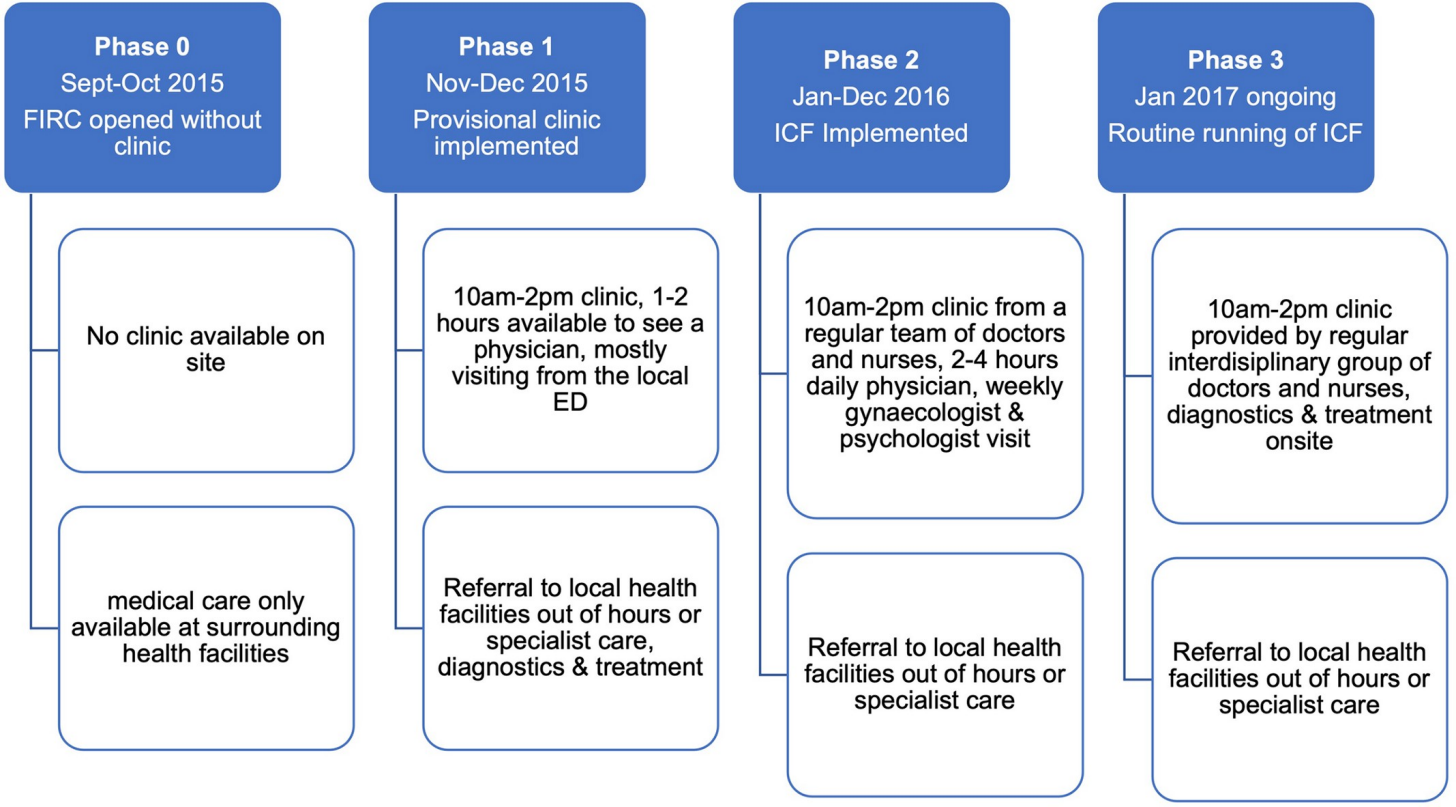
Implementation phases of the ICF.

We developed an integrated care model, that comprises of primary care for adults and children as well as specialised care (gynaecology, psychosomatic medicine and psychotherapy, and infectious diseases) within an integrated care facility (ICF) at the Freiburg initial reception centre. This vertical delivery model focuses on primary care led by a general physician and a general paediatrician, which has previously been described [23]. During the study period, the ICF was located in two consultation rooms with necessary equipment in demountable buildings inside the centre, however, was moved to a renovated building within the FIRC in 2020. Following the implementation phases, the ICF was open as a walk-in clinic on weekdays and care was provided by the two doctors assisted by two nurses for two to four hours each day (Fig 1). All medical personnel spoke both English and German, some additionally spoke French and Russian, and other interpretive support was offered on demand. Special appointments were made for maternal health questions, psychiatry (both later referred to the University Hospital), infectious disease queries and psychological concerns. Patients who were found to have other concerns covered by the University Hospital were referred there. Medical needs out of clinic hours, including weekends, were also referred to the local ED. Patient medical records were updated using the shared MeDoc software of the University Hospital in Freiburg.

#### 2.2.3 Surrounding health care providers

The biggest emergency department (ED) in the region is located at the University Hospital in Freiburg. This hospital caters to more than 50,000 patients per year, including more than 2,000 critically ill patients. The centre is equipped with specialised personnel from surgery, internal medicine, anaesthesiology and radiology.

#### 2.2.4 Study participants

The study participants included in this retrospective study are AS&R staying at the FIRC and seeking health care at either the ICF or ED. All eligible patients presenting to the ICF or ED are included in this study.

#### 2.2.5 Data sources

Individual patient data were collected retrospectively from relevant medical records of eligible study participants. A full list of variables can be found in S1. A combination of data sources were used to extract data, as data recording changed over the study period: from November 2015 until February 2016, patient data were recorded in manual format and in an Excel table; from March 2016, the MeDoc software of the University Hospital in Freiburg was used to document patient visits; from April 2018, RefCare software was introduced and for the remainder of the study period patient visits were documented in both MeDoc and RefCare prior to the official adoption of RefCare in 2019. Data were accessed on 12.02.2019, and de-identified data collected between September 2015 and June 2018 in both the FIRC and ED were imported both electronically and manually into a pre-prepared Excel spreadsheet for data analysis. The data accessed included patient demographics, language spoken by the patient and whether a translator was used, length of stay at the FIRC, frequency of visits and key reasons for their visit (including ICD-10 codes). Unfortunately, some data was not recorded for the entire study duration, for example translator presence was not recorded until March 2016.

### 2.3 Aims

First, we aim to describe absolute and relative frequency of visits to the integrated care facility from its start November 2015 to mid 2018. Secondly, we aim to analyse whether the visits and revisits are dependent on participant demographics, length of stay or translator presence. Lastly, we aim to analyse how the introduction and implementation of different phases of the integrated care model may be associated with the frequency of AS&R visits to the University Hospital’s ED.

### 2.4 Data analysis

#### Frequency of visits

To describe the absolute and relative frequency of visits per day to the ICF over time depending on reasons for seeking care, patient demographics and centre characteristics, we calculate the means and explore data using time series analyses. We apply time series methods to assess visits in relation to the different operational phases of the ICF and operational parameters at that time, specifically in relation to the staff available at the ICF, patient demographics, asylum characteristics, and translator presence. We apply an additional time series analysis using a twoway time-series line plot to explore the reasons for seeking care daily in the ICF over a period of 12 months and visualise monthly variations in relation to health needs. For each time-series graph, we used a twoway time-series line plot. The time-series was plotted to assess the frequency of daily visits (dependent variable) based on the following covariates: i) phases of the ICF and staff available, ii) key patient demographics and translator presence, and iii) the reasons for seeking care according to the recorded international classification of diseases (ICD-10). To reduce the erratic variations in the plots due to the residuals, covariates ii) and iii) were smoothed to ensure a clearer view of the time-series fit using a uniformly weighted moving average smoother, using daily lags, including the current observation, and using 62 forward terms in the filter [24]. Covariates were determined based on the quest to evaluate the impact of operational parameters, patient demographics, migrant characteristics, and reasons for seeking care on the number of daily visits, as each of these aspects may impact health care utilisation [25, 26].

#### Revisits after first visit

To describe revisits after the first visit to the ICF, we perform a survival analysis (Poisson regression) on the time for each individual since their entry into the reception centre to the point of leaving the reception centre to gain insight into AS&R seeking care and the frequency of revisits depending on demographic and operational parameters. Revisits were counted as a rate of visits per person month after the first visit to the ICF. We further fit a multivariate survival regression model and calculate the hazard ratios of returning after the first visit to assess the influence of patient demographics and asylum characteristics. For this regression, a combination of binary and categorical variables were used. Binary variables included, sex (male, female), translator presence (yes, no), duration in the FIRC (less than 6 months, greater than 6 months). Categorical variables included country of origin categories (grouped according to both region and arrival cohort), age group categories, and time of visit. Further details can be found in supplemental 1.

#### Effect of the establishment of ICF on external health care providers

To analyse the influence of the integrated care model on emergency care outside of the centre, we describe the absolute and relative frequency of visits in visits per day over time in relation to operational parameters and implementation phases. We additionally fit a time series regression to identify the impact of the implementation of the ICF on the frequency of visits to the local emergency department.

### 2.5 Ethical considerations

There are great challenges that require ethical considerations both in the work in a reception centre for AS&R as well as affiliated research. We have previously laid these out in more detail [27]. In particular we considered the vulnerable nature of the participants in addition to the question of whether the choice of setting in this case was an opportunistic one. Our argument is that a) implementation research in this vulnerable population group is very important to lead to better health services with the needs of these persons in mind, and that b) this research needs to take place in the setting where AS&R are seen and treated each day. As the data in this research was sourced from de-identified medical records, the need for participant consent was waived by the ethics committee. Ethical approval was given by the ethical committee of the Freiburg University for this study (N 271/17–273/17).

## 3. Results

### 3.1 Study population and daily frequency of visits in the ICF

**Fig 2:**
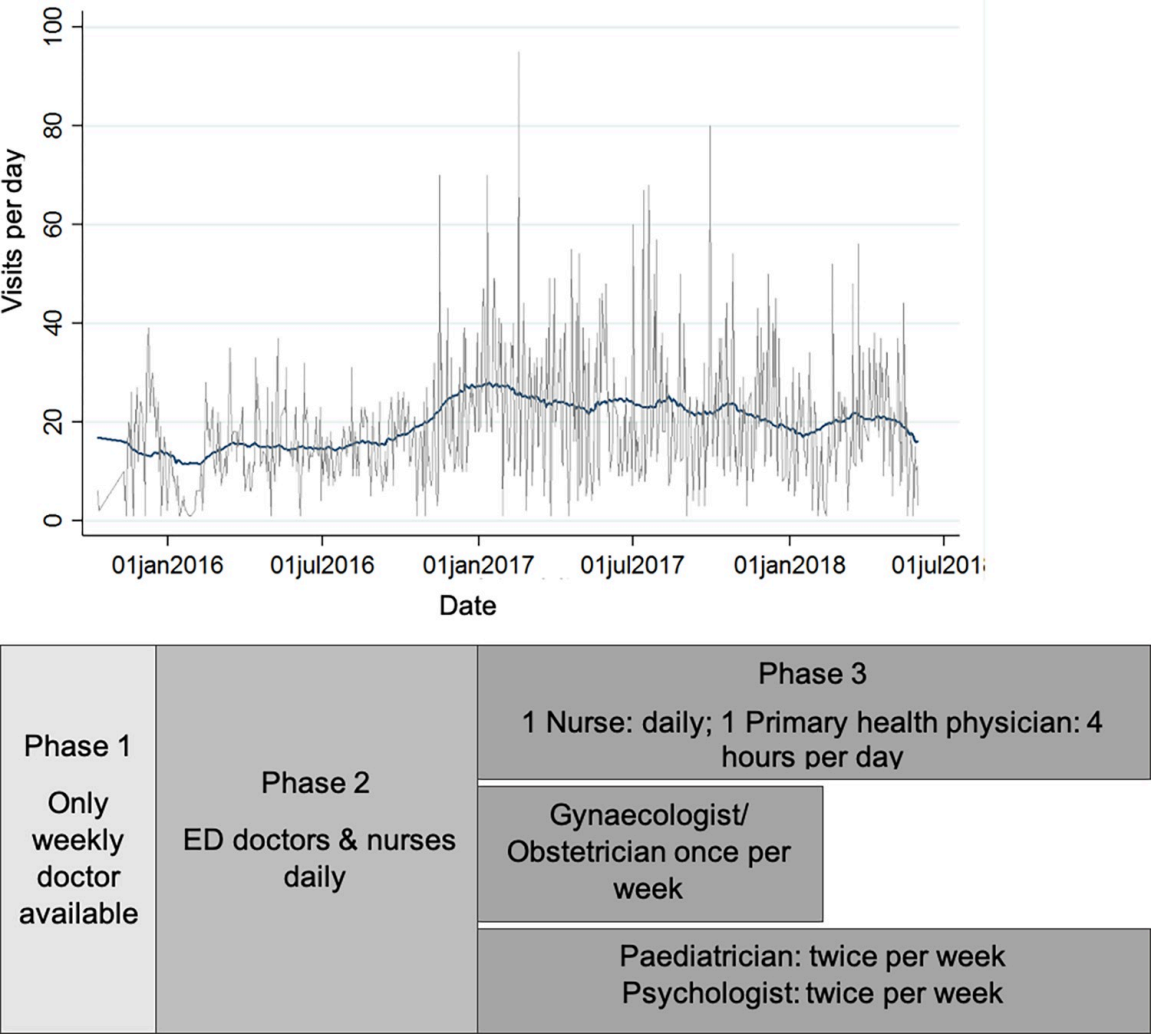
Visits per day at the ICF and staff available.

**Fig 3:**
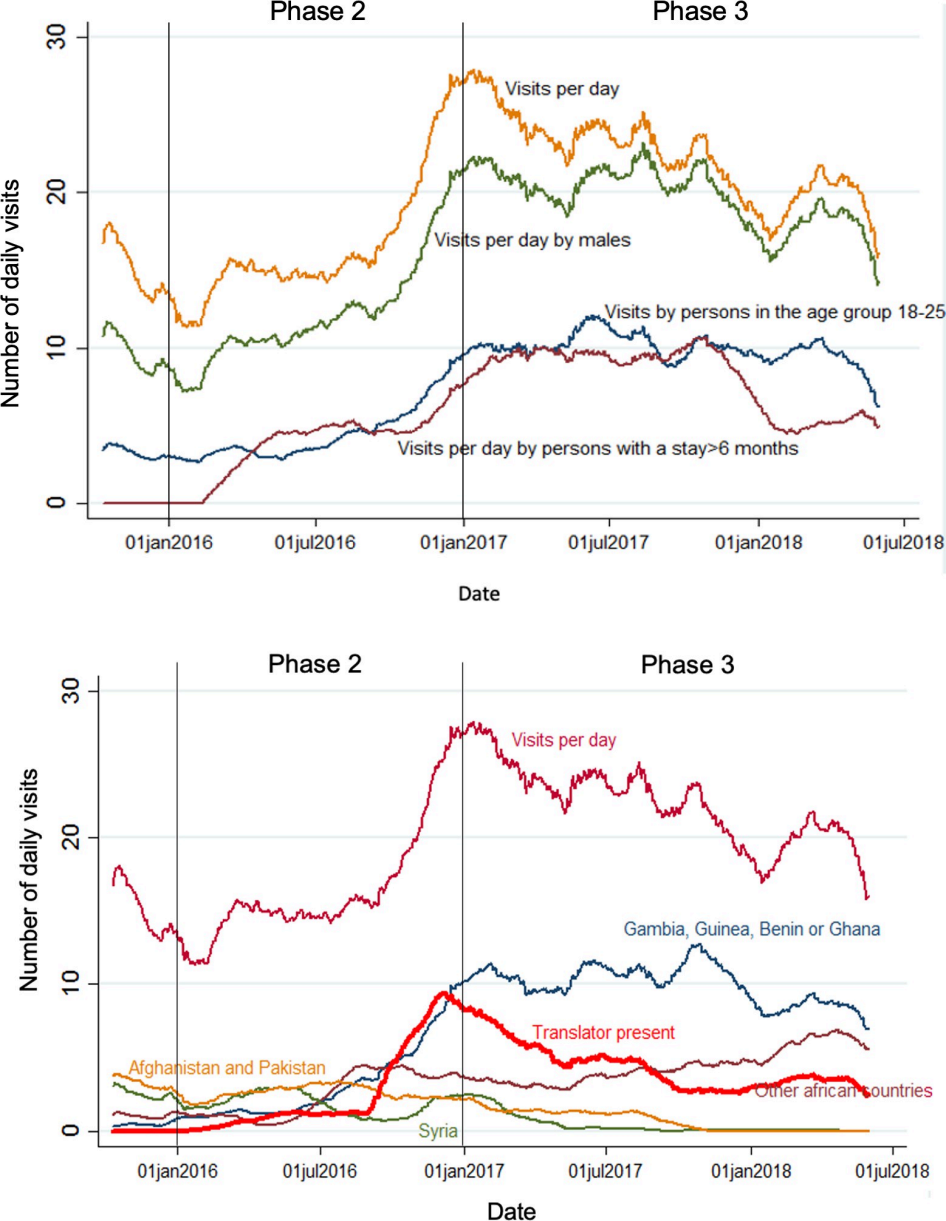
a. Demographic characteristics of visitors per day to the ICF over the study time. b. Country of origin and translator presence at visits per day at ICF.

14,419 total medical visits were counted between 11.10.2015 and 31.05.2018 from 1,892 persons seeking medical help in the ICF. Over the whole study period the mean number of persons visiting the ICF was 19·9/day (95%CI 19–20·7). The mean number of visits increased from phases 1 and 2 (implementation) to 3 (routine running), 15·8/day (95%CI 14·8–16·9) to 22·7/day (95%CI 21·4–23·9) respectively, while the proportion of visits per 100 inhabitants per day increased from 2·2 (95%CI 1·8–2·5) in phase 2 to 15 (95%CI 14–17) in phase 3 (Table 1 and Fig 2). Consistently over this study period, most inhabitants were men (Table 2), and 82% of visits were by male persons. Visit characteristics can be found in S2 Table. The 18–40 age group represented 75% of visits. However, while during 2015 and the first half of 2016 (phases 1 and 2) there was a diversity of age groups and sexes (including families with children) at the reception centre, this changed to a more homogenous group of predominantly young men from sub-Saharan Africa from July 2016 onwards (Table 2). The length of stay also changed over the study period, with an increased proportion of resident AS&R between January 2017 and January 2018 (phase 3) staying at the centre for longer than 6 months (37·4% compared to 28·7% in phases 1 and 2) (see also Fig 3A). In terms of country of origin, during 2015 and the first half 2016, phases 1 and 2, these were diverse with only minority of visitors to the ICF coming from African countries and more coming from Syria, Iran as well as the Balkan Countries, this changed from mid-2016, when a majority of West- and Central African AS&R came to the reception centre (see Fig 3B). During a similar period, the presence of translators available for the ICF was increased and more visits to the ICF happened with some form of interpretation (see Fig 3B).

**Table 1:** Mean N of visits to ICF and the emergency department over implementation phases.

	N mean number of visits to ICF	Ratio of visits to ICF per day per 100 inhabitants (95%CI)	N mean number of visits to emergency room	Ratio of visits to emergency room per day per 100 inhabitants (95%CI)
No ICF (Sep-Oct 2015)		-	2·4 (1·9–2·9)	0·28 (0·23–0·34)
Implementation ICF (Nov 2015-Dec 2016)	15·8 (14·8–16·9)	2·2 (1·8–2·5)	1 (0·6–1·4)	0·14 (0·08–0·20)
Routine running ICF (from Jan 2017)	22·7 (21·4–23·9)	15 (14–17)	0·3 (0·2–0·35)	0·29 (0·22–0·37)

**Table 2:** Patient characteristics.

	All Phases	Phase 1 Nov-Dec 2015	Phase 2 Jan-Dec 2016	Phase 3 Jan 2017- Jun 2018
Age, n	1892	306	685	901
median (IQR)	24·00 (19·07–31·27)	25·43 (18·95–34·11)	23·11 (16·88–30·89)	24·19 (19·71–31·19)
Sex, n	1888	307	681	900
male, n (%)	1464 (77·54%)	214 (69·71%)	486 (71·37%)	764 (84·89%)
female, n (%)	424 (22·46%)	93 (30·29%)	195 (28·63%)	136 (15·11%)
Translator (any)*, n	970	missing	225	745
no, n (%)	694 (71·55%)	missing	126 (56·00%)	568 (76·24%)
yes, n (%)	276 (28·45%)	missing	99 (44·00%)	177 (23·76%)
Region of origin**	1699	191	629	879
East Asia & Pacific	46 (2·71%)	0 (0·00%)	21 (3·34%)	25 (2·84%)
Europe & Central Asia	148 (8·71%)	0 (0·00%)	7 (1·11%)	141 (16·04%)
Latin America & Caribbean	0 (0·00%)	0 (0·00%)	0 (0·00%)	0 (0·00%)
Middle East & North Africa	490 (28·84%)	102 (53·40%)	274 (43·56%)	114 (12·97%)
North America	0 (0·00%)	0 (0·00%)	0 (0·00%)	0 (0·00%)
South Asia	271 (15·95%)	59 (30·89%)	137 (21·78%)	75 (8·53%)
Sub-Saharan Africa	744 (43·79%)	30 (15·71%)	190 (30·21%)	524 (59·61%)

*here, translator includes any form of interpreter, both informal and formal, and was recorded from March 2016 onwards

**regions according to World Bank analytical grouping [28]

### 3.2 Reasons for visiting the ICF

**Fig 4:**
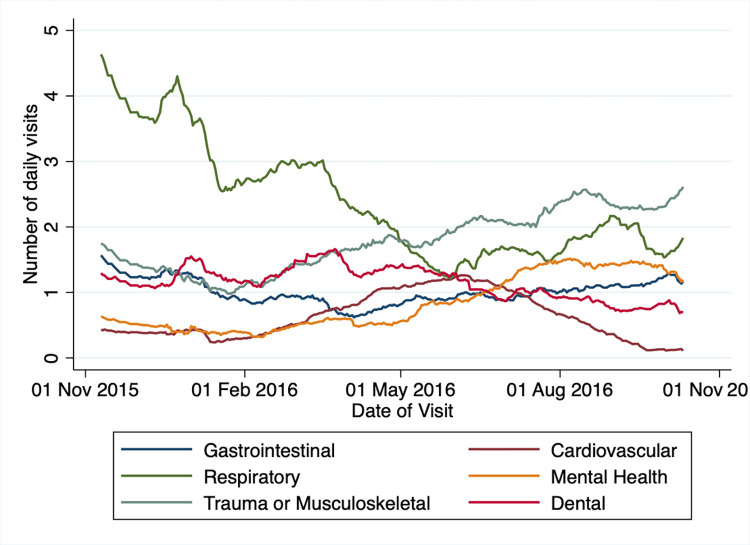
Reasons for visiting the ICF from November 2015 until November 2016.

Reasons for visiting the ICF were recorded between the end of 2015 and the beginning of 2017. During this time the majority (612/3080, 20%) of visits were due to respiratory infections and most visits due to this happened from October 2015 to January 2016 and September 2016 to October 2016. Other main reasons to visit the ICF were due to trauma and musculoskeletal conditions (441/3080, 14%), dermatological (335/3080, 11%) and dental (276/3080, 9%). Visits for musculoskeletal conditions were more frequent in summer and autumn of 2016 than at other times, while visits for respiratory illnesses were less frequent during the warmer months (see Fig 4).

### 3.3 Revisits after first visit

Overall, 10,818 visits were followed by at least one revisit or several revisits during 3,700 person months. The rate of revisits was 2·9/person month (95%CI 2·9–3). We fitted a multivariate survival regression model. Rate of revisits were decreased by male gender, younger age, originating from China, Syria or India. Rate of revisits were also decreased by length of stay >6 months, yet increased during later implementation phases (from April 2016 onwards), and by translator presence (see Table 3).

**Table 3:** Factors influencing the hazard of returning after the first visit*.

Variable		Adjusted HR
Sex	Male	0·87 (0·8–0·93
	Female	1
Age	<10	0·94 (0·82–1·1)
	10–17	0·77 (0·64–0·92)
	18–24	1
	25–40	1·24 (1·18–1·31)
	>40	1·6 (1·4–1·7)
Country of origin categories**	Gambia/Guinea/Benin/Ghana	1
	Afghanistan & Pakistan	0·7 (0·6–0·9)
	Iraq/Iran/Libya	1·2 (1·1–1·4)
	Syria	0·8 (0·7–1)
	Other African countries	1·1 (1–1·2)
	Russia/Georgia/Armenia/Serbia	0·9 (0·8–1)
	China	0·4 (0·3–0·5)
	North Africa (excluding Libya)	1·6 (1·4–1·8)
	Sri Lanka/India	0·6 (0·5–0·7)
	Turkey	1·1 (0·95–1·3)
	Other	1·5 (1·1–2)
Translator presence^#^	No	1
	Yes	1·25 (1·15–1·34)
Length of stay	0–6 months	1
	>6 months	0·82 (0·77–0·86)
Time of visit	Nov 2015 –April 2016	1
	May–Dec 2016	1·8 (1·3–2·5)
	after Jan 2017	1·7 (1·2–2·3)

*results from fitting a multivariate survival regression model of 10,818 visits during 3,700 person months

** country of origin categories are based on regions and arrival cohorts (see supplementals)

^#^ translator presence was recorded from March 2016 onwards.

### 3.4 Relative frequency of visits during different implementation phases of the ICF

The mean number of visits per 100 AS&R staying at the reception centre per day was 15 (95%CI 13–17) to the ICF and 0·28 (95%CI 0·22–0·33) to the local ED. Assessing three different implementation phases (no ICF, implementation of ICF and routine running of ICF) the ratio of visits to the local ED changed from 0·3/100 AS&R per day to 0·14/100 AS&R after implementation of the ICF. However, during the routine running, with fewer inhabitants in the reception centre, the ratio of visits increased again to 0·3/100 AS&R, while the absolute number of visits to the ED decreased consistently over time (Table 1). A time series regression could not identify a significant influence of the implementation of ICF on the number of visits to the ED in this setting.

## 4. Discussion

### 4.1 Key results

During the first three years of implementation of the ICF, the mean absolute number of visits to the health service were 19·9/day or 15/100 inhabitants per day. The major reasons for visiting the ICF were respiratory infections, trauma and musculoskeletal complaints. After having visited the ICF, inhabitants tended to visit again 2·9 times per month of further stay. Revisits were decreased by length of stay >6 months, and increased by translator presence, African origin and female sex. Visits to the main ED in Freiburg by resident AS&R halved during the implementation of ICF but increased again during routine running of the ICF.

### 4.2 Interpretation and limits of results

Health care of AS&R in the first few months after arrival is a challenging task for health care providers. While a healthy migrant effect is often discussed, potentially driven by the tendency for a younger demography of migrants that may be considered a healthier population compared to the native population [29], this often-cited healthy migrant effect is less applicable to a population that has spent months in often unsafe and unhealthy as well traumatising trajectories, not considering the reasons for fleeing to Germany.

There is a genuine lack of guidelines available to assist in developing appropriate migrant sensitive health care facilities, in particular for recently arrived AS&R [19, 20, 30]. The World Health Organisation has established some competency standards for health workers [31], and encourages migrant-sensitive care [32], which may include interpreting services, culturally relevant support and information materials, migrant health education for staff, and sufficient linkage between health care institutions to promote continuity of care [33]. While evidence indicates that interpreting services can reduce barriers to accessing care [34], and that migrant-sensitive education and improved linkage to care may improve patient outcomes [35–37], concrete framework is lacking, and more scientific evidence is required to assess the impact of migrant-sensitive health care interventions [30]. Furthermore, standards or milestones on how to organise AS&R health care have not been performed in Germany [38]. This leads to often uncoordinated practices in establishing interventions that are not evidence based [39–41].

Recently several reports of different approaches to health care for AS&R within Germany exist. In Munich, an acute situation led to an overwhelmed health care provision that was mainly bridged by relying on volunteer medical staff [42]. In Hamburg a varied approach takes care of refugees in mobile and non-mobile units within the first arrival centres [43]. Similar approaches are taken in Nuremberg and in Bremen [44]. A Heidelberg study examined the impact of a walk-in clinic in a reception centre on ambulatory care sensitive hospitalisations between 2015 and 2017, finding that such clinics may be effective in reducing hospitalisations [45].

In our setting, establishment of the ICF led only intermittently to a reduction of health care utilisation of the local ED. Whether it led to a more efficient and targeted use of the ED facilities is subject to further analysis. In general it has been described that AS&R in Germany tend to utilise ED settings more, yet less targeted than regularly insured patients [46]. Yet it must be noted that the introduction of the ICF is likely to have directed more care to the University Hospital over other medical facilities in the area, which may contribute to continual utilisation of the local ED in the routine running of the ICF. In addition, the population group and situation in the FIRC changed during the phases at the ICF. Phases 1 and 2 tended to be more similar and were during the early inception phase. During these phases, a more heterogenous group were living at the reception centre, with a mixture of groups and ages, with many of Middle Eastern origin. During this period, the residents stayed for a shorter period before moving on to other housing. This differed to the population during phase 3, the routine running of the ICF, whereby the population was more stable and made up of residents who were largely African males with considerable health needs; both physical and mental. Given the different lengths of stay and country of origin of the residents, it is also possible that the group in phases 1 and 2 were granted asylum more rapidly, while the cohort from phase 3 endured a more complicated application process and therefore were legally required to remain in the centre for a longer period. Although difficult to confirm, such a process is likely to have detrimental impacts on the mental health of residents. Subsequently, it is likely that this situation is somewhat responsible for the large visitations in this phase, and the minimal change recorded for ED visits during this period. Nonetheless, the type of care provided in the ICF differed substantially to ED; while the setting and services offered were smaller, the ICF was more targeted for migrants and their unique health needs.

Overall, health care utilisation at the ICF was slightly higher compared to other assessments [47]; pointing towards a high need for health care in the inhabitant group in the Freiburg initial reception centre. This finding is in line with a 2017 cross-sectional study of the ICF, whereby self-reported use of health care, was higher in the ICF compared to their reported use of other health care services [21]. Nevertheless, the high rate of revisits may also be due to the decreasing physician/nurse ratio as part of a watch and wait strategy and low visit threshold, combined with high nurse engagement and ease of access. Interestingly, while the mean number of visits per resident increased over time, particularly during times of low occupancy of the reception centre, the absolute number of visits did not change considerably. This may be indicative of a more severe triage effect during times of high occupancy, though familiarity with the medical staff is also likely to contribute. High rates of patient satisfaction may have contributed to this finding, as reported in the 2017 cross-sectional study[21]. However, the study also reported lower satisfaction by females and those in need of a translator [21], whom we found to have higher visits. Higher visits here may have been necessary to provide optimal care, as communication barriers could have prevented more efficient and effective responses to health needs, which also explains the reduced satisfaction. Nevertheless, translator presence increased both satisfaction and visits, and as language barriers are well documented challenges to health care for migrants, this service should be prioritised in the provision of health care for AS&R [48].

With regard to reasons for seeking care, our findings are similar to another assessment of refugees in Cologne, which found the range of reasons for visits similar to regularly insured population in Germany, particularly with respiratory infections being of concern [48].

Unfortunately, the changing formats of patient records and subsequent manual and digital data collection may have impacted our results. Due to the differing formats, some data was missing in specific periods; translator presence was missing from Phase 1, while reason for visiting the ICF was recorded differently in the changing formats. Our findings are further limited by the lack of control group and the fact that after establishment of the ICF, any critical patients were referred to the ED in Freiburg and not any of the other emergency facilities in the area. Thus, the utilisation of the ED after the establishment of the ICF may be an overestimation. It must also be noted that the use of time-series and multivariate survival analyses pose some limitations in comprehensively evaluating and interpreting our data. Such restrictions are not only due to seasonality and limitations due to selection of lags [49], but are inherent in our cohort, such as the fluctuating population of AS&R staying at the FIRC. As such, it is difficult to fully eliminate the cohort effects from the temporal trends. Though it is hard to predict when changes in the population will occur, by analysing the population in the three phases, describing the varying demographics, and exploring the health care utilisation of different groups, we are still able to grasp their impact to identify changes and prepare adaptable and suitable migrant sensitive care. Furthermore, we believe our findings are not only relevant to the situation in Freiburg during this period, but are applicable to understanding the impact of operational parameters and different patient characteristics in different settings, which could help to inform health care guidelines for AS&R. Nevertheless, more studies are required on this topic to enable greater evaluation and reduce some of the drawbacks of analyses possible with available data.

Currently, no guidelines in Germany exist around the operational standards of health care in the early care for incoming AS&R. While two reports exist looking at health care utilisation in similar circumstances [47, 48], to our knowledge this is the first assessment looking at surrounding providers in Germany as well as integrating a cohort approach with longitudinal data to estimate revisits of inhabitants. We believe that operational parameters such as the ones published here can lead to developing clearer guidelines and instructions on what health care capacity is needed when establishing health care services in reception centres.

## 5. Conclusions

This study shows that the dynamic composition of the AS&R population profoundly influences utilisation of the ICF, though seasonal factors and referral practices have an impact. While the recorded high rate of revisits may be due to the decreasing physician/nurse ratio and watch and wait strategy, ease of access and familiarity with the ICF staff may be responsible for the increase in visits despite the decline of AS&R staying at the reception centre, along with a more stable population with considerable health needs. Overall, the changes in health care use in the ICF highlight the need for a flexible model that can be readily adapted to the composition of the AS&R population. While it is vital that well-trained and experienced physicians and nurses provide this care, the discordance between the exact population of AS&R living at the reception centre and the proportion accessing care shows that the population figure alone cannot specify the number of medical staff needed. Instead, though challenging, adaptability must be prioritised to allow for frequently changing circumstances, and the availability of translators must be ensured.

## Supporting information

S1 Checklist (DOCX)

S1 Table Variables. (PDF)

S2 Table Visit characteristics. (PDF)
