# Supplementary material for: Health care utilisation of asylum seekers and refugees in the South-West of Germany
Source: PLoS One. 2024 Apr 18;19(4):e0299886. doi: 10.1371/journal.pone.0299886 (PMC11025777; doi:10.1371/journal.pone.0299886)
Supplement: S1 Table — (PDF) [file pone.0299886.s002.pdf]

S1

Variables

Collected variables

Age (continuous and categorical variable)
Sex
Country of origin
Language spoken
Translator presence
Length of stay in FIRC
Date of visits
Frequency of visits
Reasons for visits
Date of arrival
Date of departure

Variable groupings

Region of origin*

- East Asia & Pacific
- Europe & Central Asia
- Latin America & Carribbean
- Middle East & North Africa
- North America
- South Asia
- Sub-Saharan Africa

* according to World Bank analytical grouping (1)

Country of origin categories (Multivariable survival regression) Table 3$

- Other lands
- Afghanistan & Pakistan
- Gambia, Guinea, Benin & Ghana
- Iraq, Iran & Libya
- Tunisia, Algeria & Morocco
- Sri Lanka & India
- Turkey
- Other African countries: Eritrea, Nigeria, Somalia, Togo

$These groupings are based on both regions, as well as incoming cohorts of asylum seekers. For example, during phase 3, a large cohort of asylum seekers arrived from Gambia, Guinea, Benin and Ghana, while during Phase 1 and, a cohort of asylum seekers from Iraq, Iran and Libya stayed at the FIRC. This is likely due to the Königsstein key system and the EASY quota system for the distribution of asylum seekers. In this system, newly arrived asylum seekers are distributed amongst German Federal States according to a set quota based on the population and tax revenue of the State(2). Allocation of the reception centre is further

based on the country of origin and family composition; a political decision that allows for more streamlined interview processes in the regional centres in the asylum process (2).

Age categories
<10 years of age
10-17 years of age
18-24 years of age
25-40 years of age
>40 years of age

References

1. Bank W. The World by Region The World Bank 2018 [cited 2022 26.09.2022]. Available from: https://datatopics.worldbank.org/world-development-indicators/images/figures-png/world-by-region-map.pdf.
2. BAMF. Initial Distribution of asylum seekers (EASY). In: Protection AaR, editor.: BAMF; 2022.
