# Supplementary material for: Health care utilisation of asylum seekers and refugees in the South-West of Germany
Source: PLoS One. 2024 Apr 18;19(4):e0299886. doi: 10.1371/journal.pone.0299886 (PMC11025777; doi:10.1371/journal.pone.0299886)
Supplement: S2 Table — (PDF) [file pone.0299886.s003.pdf]

S2 Table
Visit characteristics

	All Phases	Phase 1<br>Nov-Dec 2015	Phase 2<br>Jan-Dec 2016	Phase 3<br>Jan 2017- Jun 2018
Age, n	14 329	646	3979	9704
median (IQR)	25·37 (19·79–32·69)	25·91 (18·65–35·84)	25·64 (18·72–33·70)	25·00 (19·97–32·05)
Sex, n	14 359	662	3993	9704
male, n (%)	11 957 (83·27%)	441 (66·62%)	2962 (74·18%)	8554 (88·15%)
female, n (%)	2 402 (16·73%)	221 (33·38%)	1031 (25·82%)	1150 (11·85%)
Translator (any)*, n	10 639	missing	1758	8881
no, n (%)	8061 (75·77%)	missing	1109 (63·08%)	6952(78·28%)
yes, n (%)	2578 (24·23%)	missing	649 (36·92%)	1929 (21·72%)
Region of origin**	13925	414	3843	9668
East Asia & Pacific	283 (2·03%)	0 (0·00%)	104 (2·71%)	179 (1·85%)
Europe & Central Asia	1297 (9·31%)	0 (0·00%)	77 (2·00%)	1220 (12·62%)
Latin America & Caribbean	0 (0·00%)	0 (0·00%)	0 (0·00%)	0 (0·00%)
Middle East & North Africa	3140 (22·55%)	204 (49·28%)	1586 (41·27%)	1350 (13·96%)
North America	0 (0·00%)	0 (0·00%)	0 (0·00%)	0 (0·00%)
South Asia	1586 (11·39%)	147 (35·51%)	756 (19·67%)	683 (7·06%)
Sub-Saharan Africa	7619 (54·71%)	63 (15·22%)	1320 (34·35%)	6236 (64·50%)
Reason for visiting[ß]	3080	678	2402	missing
Gastrointestinal	262 (8·51%	71 (10·47%)	191 (7·95%)	missing
Trauma/Musculosceletal	441 (14·32%)	69 (10·18%)	372 (15·49%)	missing
Cardiovascular	153 (4·97%)	19 (2·80%)	134 (5·58%)	missing
Dental	276 (8·96%)	50 (7·37%)	226 (9·41%)	missing
ENT/Ophtalmology	130 (4·22%)	37 (5·46%)	93 (3·87%)	missing
Pregnancy	87 (2·82%)	36 (5·31%)	51 (2·12%)	missing
Respiratory	612 (19·87%)	196 (28·91%)	416 (17·32%)	missing
Vaccination	201 (6·53%)	8 (1·18%)	193 (8·03%)	missing
Other reason	103 (3·34%)	61 (9·00%)	42 (1·75%)	missing
Dermatological	335 (10·88)	60 (8·85%)	275 (11·45%)	missing
Psychiatrical	207 (6·72%)	26 (3·83%)	181 (7·54%)	missing
Diabetes	73 (2·37%)	9 (1·33%)	64 (2·66%)	missing
Neurological	106 (3·44%)	15 (2·21%)	91 (3·79%)	missing
Urogenital	94 (3·05%)	21 (3·10%)	73 (3·04%)	missing

*translator includes any form of interpreter, both informal and formal, and was recorded from March 2016 onwards
**regions according to World Bank analytical grouping
[ß]Reason for visiting not extracted for Phase 3, due to different recording format
